# Butterfly glioblastoma: Clinical characteristics, treatment strategies and outcomes in a population-based cohort

**DOI:** 10.1093/noajnl/vdac102

**Published:** 2022-07-01

**Authors:** Line Sagerup Bjorland, Kathinka Dæhli Kurz, Øystein Fluge, Bjørnar Gilje, Rupavathana Mahesparan, Hege Sætran, Anastasia Ushakova, Elisabeth Farbu

**Affiliations:** Department of Oncology, Stavanger University Hospital, Stavanger, Norway; Department of Clinical Medicine, University of Bergen, Bergen, Norway; Stavanger Medical Imaging Laboratory (SMIL), Department of Radiology, Stavanger University Hospital, Stavanger, Norway; Institute for Data- and Electrotechnology, Faculty of Science and Technology, University of Stavanger, Stavanger, Norway; Department of Oncology and Medical Physics, Haukeland University Hospital, Bergen, Norway; Department of Clinical Science, University of Bergen, Bergen, Norway; Department of Oncology, Stavanger University Hospital, Stavanger, Norway; Department of Clinical Medicine, University of Bergen, Bergen, Norway; Department of Neurosurgery, Haukeland University Hospital, Bergen, Norway; Department of Pathology, Haukeland University Hospital, Bergen, Norway; Biostatistics, Stavanger University Hospital, Stavanger, Norway; Department of Clinical Medicine, University of Bergen, Bergen, Norway; Department of Neurology, Stavanger University Hospital, Stavanger, Norway

**Keywords:** butterfly glioblastoma, bihemispheric glioma, 3D volumetric, survival, clinical outcomes

## Abstract

**Background:**

Butterfly glioblastoma is a rare subgroup of glioblastoma with a bihemispheric tumor crossing the corpus callosum, and is associated with a dismal prognosis. Prognostic factors are previously sparsely described and optimal treatment remains uncertain. We aimed to analyze clinical characteristics, treatment strategies, and outcomes from butterfly glioblastoma in a real-world setting.

**Methods:**

This retrospective population-based cohort study included patients diagnosed with butterfly glioblastoma in Western Norway between 01/01/2007 and 31/12/2014. We enrolled patients with histologically confirmed glioblastoma and patients with a diagnosis based on a typical MRI pattern. Clinical data were extracted from electronic medical records. Molecular and MRI volumetric analyses were retrospectively performed. Survival analyses were performed using the Kaplan–Meier method and Cox proportional hazards regression models.

**Results:**

Among 381 patients diagnosed with glioblastoma, 33 patients (8.7%) met the butterfly glioblastoma criteria. Median overall survival was 5.5 months (95% CI 3.1–7.9) and 3-year survival was 9.1%. Hypofractionated radiation therapy with or without temozolomide was the most frequently used treatment strategy, given to 16 of the 27 (59.3%) patients receiving radiation therapy. Best supportive care was associated with poorer survival compared with multimodal treatment [adjusted hazard ratio 5.11 (95% CI 1.09–23.89)].

**Conclusion:**

Outcome from butterfly glioblastoma was dismal, with a median overall survival of less than 6 months. However, long-term survival was comparable to that observed in non-butterfly glioblastoma, and multimodal treatment was associated with longer survival. This suggests that patients with butterfly glioblastoma may benefit from a more aggressive treatment approach despite the overall poor prognosis.

**Key Points**

- Outcome from butterfly glioblastoma was poor with a median survival of 5.5 months.
- Long-term survival was comparable to results from general glioblastoma cohorts.
- Patients may benefit from a more aggressive treatment approach despite an overall poor prognosis.

**Importance of the Study**

Butterfly glioblastoma is a sparsely described subgroup of glioblastoma, with treatment controversies related to the poor prognosis. Results from the clinical trials on general glioblastoma may not apply to this subgroup, as patients with the most severe clinical courses are often excluded from clinical trials. This real-world study provides data on prognostic factors, treatment of, and survival from an unselected butterfly glioblastoma cohort, regardless of age and performance status. Furthermore, the population-based design ensures the identification of all residents in the region diagnosed with butterfly glioblastoma, serving as a supplement to previous retrospective observational studies arising from tertiary referral centers. Although biopsy is the diagnostic gold standard, this study demonstrates that in a real-world setting, not all patients with deep-seated brain tumors are considered amenable to an invasive procedure. This study emphasizes the importance of improving non-invasive diagnostic tools in neuro-oncology.

Butterfly glioblastoma is a subgroup of glioblastoma where a tumor crosses the corpus callosum and affects both hemispheres.^1,2^ Previous studies have reported diverging incidence rates, ranging from 2.2 to 14.3% of adults diagnosed with glioblastoma.^3–5^ It has been suggested that butterfly glioblastoma is clinically distinct from non-butterfly glioblastoma, being associated with less aggressive treatment, larger tumor volumes, and a poorer outcome.^3^ It is uncertain whether the poorer prognosis is a consequence of intrinsic tumor features or due to the choice of treatment strategies. Improved knowledge on prognostic factors and treatment, and their impact on the outcome, is required to enhance clinical decision-making. Few previous publications have described the treatment and outcome of butterfly glioblastoma.^3–7^ The optimal treatment approach remains an unresolved concern, as it is uncertain if results from studies on glioblastoma, in general, are applicable to this subgroup. A few studies have suggested a benefit from an aggressive treatment approach despite the poor prognosis.^3,4,7^ There is no definitive consensus on recurrent glioblastoma treatment.^8^ To the best of our knowledge, treatment of recurrent butterfly glioblastoma has not been described previously.

Isocitrate dehydrogenase (IDH) mutations are positive prognostic factors in glioma, but according to the 2021 WHO classification of tumors of the central nervous system, IDH-mutated astrocytoma will no longer be classified as glioblastoma.^9,10^*O*(6)-methylguanine-DNA methyltransferase (MGMT) promoter methylation is a favorable prognostic factor in glioblastoma.^11^ BRAF mutation is uncommon in glioma, but is regularly seen in epithelioid glioblastoma.^12^ Molecular characteristics of butterfly glioblastoma have only been described in small subsets in a few studies, and the prognostic value in this subgroup is not clarified.^4,7^

Although histopathological analysis is the diagnostic gold standard, not all patients are considered to be amenable to invasive diagnostics. This especially applies to patients with deep-seated butterfly tumors that are less accessible for biopsy, and severely disabled or frail patients with extensive disease at the time of diagnosis. Furthermore, histological samples are occasionally inconclusive, seen in approximately 4–6% of biopsies from brain lesions.^13–15^ Advanced MR imaging manage to differentiate high-grade glioma from other intracranial lesions with high accuracy.^14,16,17^ Due to the deep-seated tumor location of butterfly glioblastoma, MRI may also serve as a useful noninvasive prognostic tool and help to determine the preferred treatment strategy. A few previous studies have described MRI volumetric analyses in butterfly glioblastoma.^3–6^

We aimed to evaluate the prognostic values of clinical, molecular, and radiological characteristics, including volumetric analyses of T1-weighted and T2-weighted and/or fluid-attenuated inversion recovery (FLAIR) MRI, and to identify real-world treatment strategies and outcome from butterfly glioblastoma.

## Material and Methods

### Design and Sample

We conducted a population-based, retrospective cohort study with a follow-up of 7 years, or until death. We identified all patients diagnosed with glioblastoma in Rogaland and Vestland counties of Western Norway, including ten secondary and tertiary referral centers, between 01/01/2007 and 31/12/2014. We defined a butterfly tumor as a contrast-enhancing tumor crossing the corpus callosum and affecting both hemispheres, corresponding to the comparable studies.^3,4,7^ Patients with an MRI pattern meeting the butterfly tumor criteria were eligible for inclusion. Both the patients with histologically confirmed glioblastoma and patients where the diagnosis was based solely on typical MRI patterns were included, in order to avoid a selection bias by ignoring patients with extensive disease. To reduce the risk of incorrectly including patients not having a glioblastoma, we included only patients where both neuro-radiologist and clinicians considered glioblastoma the most likely diagnosis, and where differential diagnoses were considered unlikely.

### Measures

Patient characteristics and clinical findings were extracted from electronic medical records. In cases where it was not documented, Karnofsky Performance Status (KPS) was retrospectively determined based on the evaluation by nurses, physical therapists, occupational therapists, oncologists, and/or neurologists documented in the electronic medical records. We considered KPS a dichotomous variable with a cut-off value of 70 or more. Comorbidity burden was retrospectively assessed using Charlson comorbidity score.^18^ MRI reports were obtained from electronic medical records. Main tumor location was based on MRI reports in the non-butterfly glioblastoma cohort, and on volumetric analyses in the butterfly glioblastoma cohort. We compared clinical characteristics and treatment modalities in patients diagnosed with butterfly glioblastoma and patients diagnosed with non-butterfly glioblastoma in the same region and study period (Table 1).

**Table 1.** Clinical characteristics and treatment modalities in 360 patients diagnosed with butterfly glioblastoma (*n* = 33) or non-butterfly glioblastoma (*n* = 327) in Western Norway between 01/01/2007 and 31/12/2014

	Butterfly glioblastoma (*n* = 33)	Non-butterfly glioblastoma (*n* = 327)	*P* value^a^
Patient characteristics			
Female	20 (60.6%)	131 (40.1%)	.023
Age ≥ 70 years	13 (39.4%)	114 (34.9%)	.60
Age, median (range)	66.6 (27.9–84.8)	64.6 (18.1–94.9)	.66
Karnofsky performance status < 70^b^	8 (24.2%)	N/A	–
Charlson Comorbidity Score ≥ 7	5 (15.2%)	36 (11.1%)	.56
Symptom(s) at time of diagnosis			
Cognitive impairment	23 (69.7%)	148 (45.3%)	.007
Headache	11 (33.3%)	146 (44.6%)	.21
Epilepsy	6 (18.2%)	102 (31.2%)	.12
Paresis	8 (24.2%)	112 (34.3%)	.25
Dizziness	7 (21.2%)	55 (16.8%)	.52
Central facial palsy	4 (12.1%)	92 (28.1%)	.047
Dysphasia	3 (9.1%)	82 (25.1%)	.039
Hemianopia	2 (6.1%)	52 (15.9%)	.20
MRI characteristics			
Main tumor location^c^			< .001
Frontal	9 (27.3%)	72 (22.1%)	
Temporal	8 (24.2%)	82 (25.2%)	
Occipital	8 (24.2%)	8 (2.5%)	
Deep-seated	6 (18.2 %)	31 (9.5%)	
Parietal	2 (6.1 %)	28 (8.6%)	
Overlapping	0 (0.0%)	105 (32.2%)	
Primary treatment			
Number of treatment modalities^d^			< .001
None (best supportive care)	6 (18.2%)	24 (7.3%)	
1 modality	8 (24.2%)	51 (15.6%)	
2 modalities	16 (45.5%)	75 (22.9%)	
3 modalities	4 (12.1%)	177 (54.1%)	
Resection	4 (12.1%)	215 (65.7%)	< .001
Radiation therapy	27 (81.8%)	292 (89.3%)	.16
Radiation therapy schedule^e^			.014
Hypofractionated	16 (59.3%)	103 (35.3%)	
Standard fractionated^f^	11 (40.7%)	189 (64.7%)	
Temozolomide concurrent and/or adjuvant	19 (57.6%)	225.(68.8%)	.20

Age presented absolute number (%) aged over 70 years and median (range), all other characteristics presented as absolute number (%).

MRI, magnetic resonance imaging; FLAIR, fluid attenuated inversion recovery.

^a^Comparison between groups was performed by Chi-square Test (Fisher’s exact Test when expected cell count < 5) for categorical data and Mann–Whitney *U* Test for the continuous age variable. *P* values < .05 were considered statistically significant.

^b^Not available for the non-butterfly glioblastoma cohort.

^c^For butterfly glioblastoma based on volumetric analyses, for non-butterfly glioblastoma based on MRI reports.

^d^Surgical resection, radiation therapy and chemotherapy (temozolomide).

^e^Among patients who received radiation therapy.

^f^60 Gy in 2 Gy fractions.

Further analyses were performed on the butterfly glioblastoma cohort only. Histological samples were re-evaluated by a neuropathologist (Table 2). Molecular analyses were retrospectively performed on primary tissue samples. *O*(6)-methylguanine-DNA methyltransferase (MGMT) promoter methylation status was detected using methylation-specific polymerase chain reaction (MSP) as a qualitative method.^19,20^ Determination of IDH mutational status was restricted to the identification of the most frequent mutation, IDH1 R132H, and detected by immunohistochemistry with an IDH1 R132H specific monoclonal antibody. Identification of BRAF mutational status was restricted to the identification of mutation of codon 600 of exon 15 (V600E), being the most frequent activating mutation of the BRAF gene. Sanger DNA sequencing of exon 15, sequencing both the forward and the reverse strand, was performed.

**Table 2.** Imaging and molecular characteristics in 33 patients diagnosed with butterfly glioblastoma between 01/01/2007 and 31/12/2014

MRI characteristics	
Corpus callosum affection^a^	
Rostrum/genu	21 (63.6%)
Body	25 (75.8%)
Splenium	14 (42.4%)
Tumor distribution	
Left skewed	13 (39.4%)
Right skewed	13 (39.4%)
Symmetric	7 (21.2%)
Tumor volumes	
T1-weighted contrast-enhanced MRI (cm^3^)	41 (26–73)
T2-weighted/FLAIR MRI (cm^3^)	137 (83–229)
Ratio T2/FLAIR:T1 volumes	3.2 (2.4–4.4)
Hypothalamus involvement	11 (33.3%)
Basal ganglia involvement	14 (42.4%)
Blood vessel affection	20 (60.6%)
Necrosis	28 (84.8%)
Flow void	23 (69.7%)
Mass effect^b^	16 (48.5%)
Molecular characteristics (*n* = 11)^c^	
MGMT promoter methylation status	
Inconclusive	2 (18.2%)
Methylated	2 (18.2%)
Unmethylated	7 (63.6%)
BRAF mutational status	
BRAF V600E mutation	0 (0.0%)
BRAF V600E wild type	11 (100.0%)

Tumor volumes and T2/FLAIR:T1 ratio presented as median (IQR), all others as absolute numbers (%). Tumor volumes defined as contrast enhancement and necrosis in T1-weighted MRI and tumor associated non-enhancing hyperintense lesions in T2-weighted/FLAIR MRI.

MRI, magnetic resonance imaging; FLAIR, fluid attenuated inversion recovery; cm^3^, cubic centimeters; MGMT, *O*(6)-methylguanine-DNA methyltransferase.

^a^Sum exceeding 100% due to the affection of multiple regions in several patients.

^b^Defined as midline shift ≥ 1 mm.

^c^Presented as absolute numbers and % of patients with histological sample available for re-evaluation and molecular analyses.

MR images at the time of diagnosis were re-evaluated by a neuro-radiologist, and volumetric analyses were performed (Table 2). Images were acquired from four different hospitals over an 8-year period, and imaging protocols varied regarding sequences and quality. T1-weighted series prior to, and after, intravenous gadolinium containing contrast agent, and T2-weighted and/or FLAIR series were present in all the patients. Tumor characteristics were identified (Table 1). The main location was defined as the lobe/region mainly affected by the tumor. Involvement of the corpus callosum was classified according to Highley and colleagues.^21^ Mass effect was measured as mm midline shift, and was classified as slight or severe with a cut-off value of 10 mm, in line with a comparable study.^22^ Contrast-enhancing tumors on the T1-weighted series and tumor-associated non-enhancing hyper-intense lesions on T2-weighted/FLAIR series were identified, according to standardized neuro-oncological tumor assessment.^23^ Volumes were delineated using the open-source software platform 3D slicer Version 4.10.1, and measured in cm^3^. Necrosis was included in the T1 volumes, and T1 volumes were included in the T2/FLAIR volumes. Flow void was defined as the absence of signal in the lumen of a pathological or normal-appearing cerebral artery and reflects a blood flow of significant velocity. Blood vessel affection was defined as a normal-appearing cerebral artery completely enfolded by the tumor.

Treatment and complications in primary and recurrent situations were registered (Table 3). As discontinuation or changes in treatment is a frequent concern, the completion of radiation therapy (60 Gy in 2 Gy fractions) with concurrent temozolomide administered daily during the entire period, and the completion of at least one of six planned temozolomide monotherapy courses, were classified as treatment according to the Stupp protocol.^24^ All the other combinations of radiation therapy and/or temozolomide were classified as less-intensive chemoradiotherapy. A significant number of patients had the diagnosis based on MRI, lacking a date of surgery or biopsy. To ensure a consistent determination of the time of diagnosis, we decided on defining this as the date of the first MRI presenting with the tumor. Progressive disease was defined as an unequivocal clinical or radiological progression. Long-term survival was defined as survival of 3 years or more, in line with previous studies.^25,26^

**Table 3.** Treatment and complications in 33 patients diagnosed with butterfly glioblastoma between 01/01/2007 and 31/12/2014

Primary treatment	
Surgery	
Resection	4 (12.1%)
Biopsy	11 (33.3%)
None	18 (54.5%)
Chemoradiotherapy	
Stupp protocol^a^	8 (24.2%)
Less-intensive^b^	19 (57.6%)
None	6 (18.2%)
Number of adjuvant TMZ courses	3 (1–9)
Anti-tumor treatment last 30 days of life	6 (18.2%)
Complications during follow-up	
Surgical complications^c^	2 (13.3%)
Epileptic seizure	13 (39.4%)
Venous thromboembolism	10 (30.3%)
Hematotoxicity^d^	3 (15.8%)
Osteoporosis	4 (12.1%)
Treatment at recurrence (*n* = 18)^e^	
Best supportive care	11 (61.1%)
Surgical resection	2 (11.1%)
Stereotactic radiosurgery	1 (5.6%)
Re-irradiation	0 (0.0%)
TMZ monotherapy	5 (27.8%)
CCNU-based chemotherapy (PCV^f^)	1 (5.6%)
Bevacizumab and nitrosoureas (CCNU or BCNU)	2 (11.1%)

Number of TMZ courses presented as median (range), all others as absolute numbers (%).

CTCAE, common terminology criteria for adverse events; TMZ, temozolomide; CCNU, cyclonexyl-chloroethyl-nitrosourea (lomustine); BCNU, bis (chloroethyl) nitrosourea (carmustine).

^a^Defined as the completion of radiation therapy (60 Gy in 2 Gy fractions) with concurrent TMZ and at least one of six planned TMZ monotherapy courses).

^b^Included 16 patients with hypofractionated radiation therapy with or without TMZ and three patients with standard fractionated radiation therapy with TMZ to a less extent.

^c^Complications among 15 patients who had undergone biopsy or resection included cerebral infarction and paresis (n=1) and increasing dysphasia (n=1).

^d^CTCAE ≥ grade 3 among 19 patients receiving TMZ.

^e^Numbers include 18 patients where relapse was detected, while two patients with no sign of recurrence during follow-up and 13 patients who had a continuous deterioration and died without being diagnosed with recurrence were not included.

^f^Procarbazine, Lomustine (CCNU), and Vincristine.

### Statistics

Statistical analyses were performed using Statistical Package for the Social Sciences (SPSSs) Version 16, and figures were created in R Version 4.0.4. Categorical data were presented as counts and percentages, and the between-group differences were assessed using Chi-square (*χ*^2^) and Fisher’s exact test, as appropriate. Continuous variables were presented as mean ± SD or median and range or interquartile range (IQR). Comparisons of two and multiple groups were performed using the Mann–Whitney *U* and Kruskal–Wallis tests, respectively. Correlations between continuous variables were assessed using the Pearson correlation test, and presented as Pearson correlation coefficient (*r*). Survival analyses were performed using the Kaplan–Meier plots and Cox proportional hazards regression models. To investigate the relations between patient characteristics and survival, we applied an unadjusted Cox model and a Cox model adjusted for age, sex, and KPS. No correction for multiple comparisons was made. *P*-value < .05 were considered statistically significant.

### Ethics

The study was approved by the Regional Committees for Medical and Health Research Ethics (REK Vest 2014/1931). Informed consent was obtained from patients alive at the time of inclusion. Waiver of consent was approved for deceased patients.

## Results

We identified 381 patients diagnosed with glioblastoma by typical MRI pattern or histological sample. Eighteen patients were excluded due to residence outside the region (*n* = 1), known previous low-grade or anaplastic glioma (*n* = 10, including one patient with IDH mutated butterfly glioblastoma), synchronous cancer (*n* = 4), lack of informed consent (*n* = 1), lost-to-follow-up (*n* = 1), and disproven glioblastoma diagnosis by autopsy (*n* = 1), resulting in a cohort of 363 patients that was described in a previous study.^27^ Among the 363 patients eligible for further analyses, 327 patients had a non-butterfly tumor and 36 patients had a bi-hemispheric tumor crossing the corpus callosum. Among 36 patients with butterfly tumor distribution, one patient not meeting the histopathological criteria for glioblastoma and two patients with IDH1 mutation were excluded. Finally, 33 patients (8.7% of patients diagnosed with glioblastoma) met the criteria for butterfly glioblastoma and were enrolled. Clinical characteristics and treatment modalities in patients with non-butterfly glioblastoma compared with the subgroup with butterfly glioblastoma are presented in Table 1.

Among the 33 patients with butterfly glioblastoma, the diagnosis was histologically confirmed in 13 patients (39.4 %), and for the remaining 20 patients (60.6 %) the diagnosis was based on MRI pattern only. The latter group included two patients with non-representative biopsies. Among the 13 patients with conclusive biopsies, 11 samples were available for further analyses. Radiological and molecular characteristics are presented in Table 2. Sixteen patients (48.5%) presented with a midline shift, mean 7.9 mm (SD ± 3.3), and with a severe midline shift of 10 mm or more in four patients.

**Figure 1.**
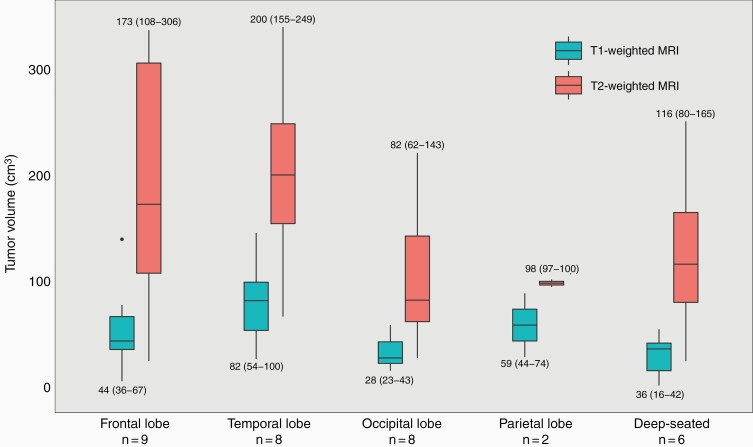
Median [interquartile range (IQR)] contrast-enhancing tumor volume in T1-weighted magnetic resonance imaging (MRI) and median (IQR) volume of tumor-associated non-enhancing hyperintense lesions in T2-weighted and/or fluid-attenuated inversion recovery (FLAIR) MRI according to tumor location in 33 patients diagnosed with butterfly glioblastoma between 01/01/2007 and 31/12/2014.

Median contrast-enhancing tumor volume in T1-weighted images was 41 cm^3^ (range 2–146). The median volume of non-enhancing hyperintense lesions in T2-weighted/FLAIR images was 137 cm^3^ (range 25–340). The ratio between T2/FLAIR and T1 volumes ranged from 1.1 to 12.5. Tumor volumes and locations are shown in Figure 1. Median T1 volumes were smallest in central and occipital tumors with median volumes of 37 cm^3^ (IQR 8–46) and 28 cm^3^ (IQR 22–48), however, without a statistical difference (*P* = .07). There was no difference in median T2/FLAIR (*P* = .11) or T2/T1 ratio (*P* = .10) between different locations.

Both median T1 volume and median abnormal T2/FLAIR volume were larger in the resection group than the non-resection group: 109 cm^3^ vs 39 cm^3^ (*P* = .005) and 281 cm^3^ vs 109 cm^3^ (*P* = .02). Larger T1 volume was correlated with younger age [*r* = ‐0.365 (*P* = .04)], while no correlation between T2 volume and age was observed [*r* = ‐0.072 (*P* = .69)]. All the three patients with the largest T1 volumes (≥ 100 cm^3^), and three of four patients with the largest T2 lesions (≥ 300 cm^3^), were aged under 70 years.

### Treatment and Survival

Treatment modalities in patients with butterfly glioblastoma compared with non-butterfly glioblastoma are presented in Table 1. Radiation therapy was given to 27 of 33 patients (81.8%) with butterfly glioblastoma and 292 of 327 patients (89.3%) with non-butterfly glioblastoma (*P* = .16). Among patients receiving radiation therapy, hypo-fractionated radiation therapy was performed in 16 of 27 patients (59.6%) in the butterfly cohort received, compared with 103 of 292 patients (35.3%) in the non-butterfly cohort (*P* = .014). Among 16 patients with butterfly glioblastoma receiving hypo-fractionated radiation therapy, the most frequently used hypo-fractionated radiation schedule was 39 Gy in 3 Gy fractions (given to 14 patients), and eight of patients (50.0%) received concomitant and/or adjuvant temozolomide

Additional data on treatment strategies and complications in the butterfly glioblastoma cohort are outlined in Table 3. Four patients (12.1%) underwent surgery in the primary situation. All these were aged under 70 years, and all had an incomplete resection with remaining contrast-enhancing residual tumor based on postoperative MRI. One patient suffered from a perioperative stroke, resulting in increasing and permanent hemiparesis, while the three others had no surgical complications. Eight patients (24.2%) received chemoradiotherapy treatment according to the Stupp protocol, while 19 patients (57.6%) underwent less-intensive chemoradiotherapy. Six patients (18.2%) were treated with the best supportive care, including four patients where hypo-fractionated radiation therapy was planned, but canceled because of a rapid clinical deterioration.

**Figure 2.**
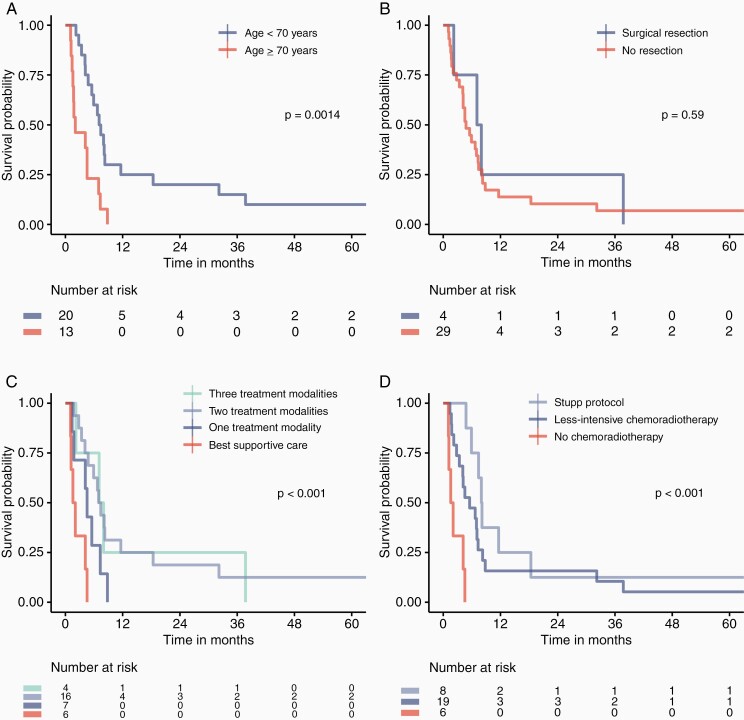
The Kaplan–Meier curves of survival probability in 33 patients diagnosed with butterfly glioblastoma between 01/01/2007 and 31/12/2014. Cumulative survival in months. Survival by (a) age, (b) surgical resection, (c) number of treatment modalities, and (d) chemoradiotherapy regimen. Comparison of groups by log rank test. *P*-values < .05 were considered statistical significant.

Median overall survival in the butterfly glioblastoma cohort was 5.5 months (95% CI 3.1–7.9), and median progression-free survival was 3.8 months (95% CI 3.0–4.7). One-, 2-, and 3-year survival rates were 15.2%, 12.1%, and 9.1%, respectively. Median survival in patients treated according to the Stupp protocol was 8.0 months (95% CI 7.0–9.0), compared with 5.5 months (95% CI 1.9–9.1) and 1.6 months (95% CI 0.5–2.6) in patients treated with less-intensive chemoradiotherapy and best supportive care (*P* < .001). Median survival in patients older than 70 years was 2.1 months (95% CI 0.0–5.0), compared with 7.1 months (95% CI 5.6–8.6 months) in patients aged under 70 years (*P* = .001). Nine patients (27.3%) died within 3 months of diagnosis. Overall survival in 13 patients with histologically confirmed butterfly glioblastoma was 8.0 months (95% CI 4.9–11.0). Survival curves are presented in Figure 2.

Older age and mainly deep-seated tumor location was associated with poor survival according to Cox regression adjusted for age, sex, and KPS, with HR 1.06 (*P* = .003) and HR 4.58 (*P* = .03), respectively. There was not revealed any associations between T1 or T2/FLAIR volumes and outcome. Best supportive care was associated with poorer outcomes compared with the multimodal treatment (HR 5.11, *P* = .04), whereas the impact from one treatment modality was not significant (HR 1.67, *P* = .46). Unadjusted and adjusted analyses are presented in Table 4.

**Table 4.** Unadjusted and adjusted analyses on overall survival in 33 patients diagnosed with butterfly glioblastoma between 01/01/2007 and 31/12/2014

Variable	Unadjusted		Adjusted for sex, age, and KPS	
	HR (95% CI)	*P* value	HR (95% CI)	*P* value
Sex				
Male	Ref.		Ref.	
Female	0.74 (0.36–1.52)	.41	0.74 (0.36–1.54)	.42
Age (per year)	1.06 (1.03–1.10)	**.001**	1.06 (1.02–1.10)	**.003**
KPS				
≥70	Ref.		Ref.	
<70	2.96 (1.21–7.21)	**.017**	1.86 (0.73–4.74)	.19
Tumor side				
Left	Ref.		Ref.	
Right	1.28 (0.57–2.86)	.56	1.51 (0.63–3.59)	.35
Equal	1.95 (0.75–5.05)	.17	1.77 (0.64–4.91)	.27
Main tumor location				
Frontal lobe	Ref.		Ref.	
Temporal lobe	3.59 (1.11–11.65)	**.034**	1.32 (0.31–5.70)	.71
Occipital lobe	1.59 (0.56–4.52)	.39	1.70 (0.58–4.98)	.33
Parietal lobe	0.43 (0.05–3.43)	.42	0.99 (0.11–9.03)	1.00
Deep-seated	6.69 (1.84–24.35)	**.004**	4.58 (1.15–18.20)	**.031**
MRI T1 volume (per cm^3^)	1.00 (0.99–1.01)	.68	1.00 (0.99–1.02)	.56
MRI T2/FLAIR volume (per cm^3^)	1.00 (1.00–1.01)	.33	1.00 (1.00–1.01)	.15
T2/FLAIR:T1 ratio (per unit)	1.01 (0.88–1.16)	.90	0.95 (0.79–1.14)	.58
Hypothalamus involvement	2.02 (0.95–4.32)	.07	1.36 (0.63–2.93)	.44
Basal ganglia involvement	1.63 (0.79–3.35)	.19	1.23 (0.54–2.83)	.62
Blood vessel involvement	0.57 (0.27–1.19)	.13	0.94 (0.40–2.23)	.89
Mass effect (per mm midline shift)	0.95 (0.87–1.04)	.24	0.98 (0.89–1.08)	.67
Necrosis	0.64 (0.24–1.71)	.38	0.37 (0.13–1.06)	.06
Flow void	0.64 (0.29–1.41)	.27	0.97 (0.40–2.35)	.95
MGMT promoter methylation status				
Unmethylated	Ref.			
Methylated	0.29 (0.04–2.42)	.25	0.06 (0.00–3.33)	.17
Primary treatment				
Two or three modalities	Ref.		Ref.	
One modality	2.37 (0.95–5.96)	.07	1.67 (0.43–6.51)	.46
Best supportive care	8.10 (2.65–24.79)	**< .001**	5.11 (1.09–23.89)	**.038**

HR, 95% CI and *P*-values calculated by unadjusted and adjusted Cox proportional hazards regression. Deep-seated location defined as tumor mainly located in thalamus, basal ganglia, capsula interna, splenium corpus callosum, or mesencephalon. Tumor volumes in cm^3^. Mass effect measured as midline shift in millimeters (mm). Volumes defined as contrast-enhancing tumor volume in T1-weighted MRI and tumor-associated non-enhancing hyperintense lesion in T2-weighted/FLAIR MRI. Only patients with conclusive result were included in MGMT analyses (*n* = 9). Treatment modalities included surgical resection, radiation therapy, and temozolomide concurrent and/or adjuvant. *P*-values < .05 were considered statistically significant.

HR, hazard ratio; CI, confidence interval; KPS, Karnofsky performance status; MRI, magnetic resonance imaging; FLAIR, fluid attenuated inversion recovery; MGMT, *O*(6)-methylguanine-DNA methyltransferase.

All the three long-term surviving patients were females younger than 70 years with good performance scores (KPS ≥ 70), and two of them had a histologically confirmed IDH1 wildtype glioblastoma. These two had both a severe midline shift, although highly different tumor volumes, with T1 volume, T2/FLAIR volume, and T2/FLAIR:T1 ratio of 11 cm^3^ vs 140 cm^3^, 108 cm^3^ vs 255 cm^3^, and 9.8 vs 1.8, respectively.

Two patients (6.1%) had stable disease with no sign of recurrence during follow-up. Thirteen patients (39.4%) had no period of improvement or stable disease after primary diagnosis, presenting with a continuous progression and deterioration until death, without being diagnosed with a recurrence. In the remaining 18 patients (54.5%), an interval with improvement or stabilization after primary treatment was observed, thereafter being diagnosed with a recurrence. Seven of 18 patients (38.9%) diagnosed with recurrence received various anti-tumor treatments (Table 3). Median post-recurrence survival was 4.1 months (95% CI 2.8–5.4) in patients receiving anti-tumor treatment, compared with 1.5 month (95% CI 0.9–2.1) in patients receiving the best supportive care (*P* = .04).

## Discussion

In this retrospective population-based cohort study, we found that 8.7% of adults diagnosed with glioblastoma met the criteria for butterfly glioblastoma. The outcome was poor, with a median overall survival of less than 6 months. This study adds real-world data on prognostic factors and outcome in a previously sparsely described subgroup of glioblastoma, whereas previous studies are based on data from tertiary referral centers.^3–5,7^

### Treatment

Hypo-fractionated radiation therapy with or without temozolomide was the most common treatment approach in the butterfly glioblastoma cohort, given to 59% of patients that were considered eligible for receiving radiation therapy. Likely, explanations include the severe disease course and poor prognosis. In a real-world setting, clinical decision-making is based on individual considerations. Hypo-fractionated radiation therapy may be an appropriate approach in this poor-prognostic subgroup, however, not standard of care in patients younger than 70 years. Ideally, it should be prospectively evaluated if hypo-fractionated radiation therapy, regardless of age, is non-inferior to standard treatment.

### Survival

Our study confirms previous findings of a considerably poorer prognosis in butterfly glioblastoma compared with glioblastoma in general, where population-based studies observed a median overall survival of nine to 11 months.^28–31^ Median overall survival of 5.5 months in our cohort was comparable with the results from previous butterfly glioblastoma studies, with median survival ranging from 3.2 to 5.9 months.^4,5,7^ Survival was substantially poorer than that observed by Burks and colleagues, where median survival was 12 and 15 months in patients who underwent two different surgical approaches.^6^ Likely explanations are different selection criteria and settings, and highly different resection rates of 12% and 100%. Although these two studies cannot be compared directly, this may indicate a potential benefit from a more aggressive treatment approach in patients with butterfly glioblastoma. Patients older than 70 years received less-intensive treatment than younger patients, and had a significantly poorer outcome with a median survival of only 2 months, equal to the findings of Dayani and colleagues.^4^ Previous studies have demonstrated that combined chemoradiotherapy improves outcome in elderly glioblastoma patients with adequate performance status.^32–34^ Future studies, preferably including quality of life analyses, may clarify if these results are applicable to the butterfly glioblastoma subgroup.

Despite the poor overall outcome, patients with long-term survival were observed in 9.1% of the patients, not markedly poorer than pooled 3-year survival rate of 11% in a large meta-analysis of glioblastoma in general.^35^ We suggest that patients with butterfly glioblastoma may receive less-intensive treatment due to the deep-seated tumor location, the uncertainty of treatment benefit, the dismal prognosis. This study demonstrates that long-term survival is possible, supporting an argument for a higher treatment intensity in patients in acceptable general conditions. A second argument for providing more multimodal treatment is the challenge of predicting treatment benefit, long-term survival, and quality of life in these patients. Only a minority of patients receiving temozolomide suffered from significant toxicity, serving as a third argument to promote an increased treatment intensity in patients with butterfly glioblastoma. Nearly 40% of the patients diagnosed with recurrent glioblastoma received anti-tumor treatment and had a median post-recurrence survival of approximately 4 months. We suggest that anti-tumor treatment may be appropriate in patients with recurrent butterfly glioblastoma and acceptable performance status.

### MR Volumetric Analyses

All the three patients with the largest tumor volumes in T1-weighted MRI, and three of four patients with the largest abnormal T2/FLAIR volumes, were aged younger than 70 years. Among possible explanations, we suggest that the younger patients, having a higher cognitive reserve, tolerate larger tumor growth before symptoms occur. Furthermore, the tumor volumes of long-term surviving patients differed widely, ranging from the upper to lower quartiles. We suggest that the larger tumor volumes may not be considered an unequivocal negative prognostic factor, as larger volumes may be seen in the younger patients harboring favorable prognostic factors. The association between T2/FLAIR volumes and age has not been studied previously, thus comparison with other studies was not applicable.

In our cohort, both median T1 and T2/FLAIR volumes were larger in the resection group than the non-resection group. Larger tumor volumes in younger patients and larger tumor volumes in frontal lobes, with favorable surgical accessibility, are possible explanations. In contrast, Dziurzynski and colleagues^5^ reported slightly larger, but statistically insignificant, median T1 volume in the non-resection group compared with the resection group, although ranges were wide in both groups and with no difference in FLAIR volumes. Among possible explanations for these differences are the different settings, small sample sizes, low-resection rates of 16% and 36%, and the lack of adjustment for molecular subgroups. Unlike Dayani and colleagues,^4^ who reported a slight negative correlation between larger T1 volume and survival, we found no such association. Small sample sizes and wide range of tumor volumes may explain this uncertainty. This study did not reveal any significant associations between T1 or T2/FLAIR volumes and survival, however, this may not be evaluable in this small sample.

### Diagnosis and Lack of Biopsy

Biopsy is the gold standard for diagnosing glioblastoma. However, a significant number of patients in our cohort had the diagnosis based on a typical MRI pattern. This pragmatic approach of restraining from a biopsy may have several explanations; butterfly glioblastomas are not easily accessible for biopsy as they are sited in deep brain structures and as traditionally butterfly glioblastomas were considered a poor prognostic group with limited treatment benefits, risk of taking biopsy might have been difficult to be justified. In addition, as these patients have limited treatment options and life expectancy, demographic factors may also have had an impact on clinical decision-making. Long distance patient transport may be a significant burden for both patients and health care services. It is likely to assume that other regions and settings worldwide experience similar challenges and controversies. As advanced MRI techniques provides a high-diagnostic accuracy, it might have been easier and feasible to rely on MRI diagnosis. However, the authors acknowledge that due to improved surgical techniques and increased importance of molecular analyses, the majority of patients with butterfly gliomas should undergo biopsy or resection in current clinical practice. Finally, there was an occasional occurrence of inconclusive CNS biopsies, also seen in other studies.^13–15^ There is a need for improved non-invasive diagnostic methods such as advanced MRI techniques.

### Strengths and Limitations

Strengths of this study included the population-based study design and that all the patients were followed up for 7 years or until death. Furthermore, the clinical information on all patients was available in a common electronic record, and T1-weighted and T2-weighted/FLAIR images were available in all the patients. The main limitation is the high lack of histological samples in our cohort. This causes a risk of inclusion bias and a high number of missing molecular data. Enrolling the patients with MRI-based diagnosis has the disadvantage of potentially including non-glioblastoma patients. However, exclusion of the same patients may lead to a systematic selection bias of patients having the most severe disease courses. We only perform the analysis of IDH1 mutation, and did not include analysis of IDH2 mutational status. Another limitation is the small sample size. However, this is related to the rarity of the condition and is comparable to the previous studies.^3–5,7^ Furthermore, there is a risk of informational bias associated with the retrospective estimation of KPS. To reduce this risk, we considered KPS a dichotomous variable, not distinguishing between exact values over and under this cut-off value of 70. Also, associations between treatment and outcome in elderly patients were inconclusive, as only a minority of elderly patients received multimodal treatment.

To conclude, outcome from the butterfly glioblastoma was dismal, with a median overall survival of less than 6 months. However, a long-term survival was comparable to that observed in glioblastoma in general, and multimodal treatment was associated with longer survival. This suggests that patients with butterfly glioblastoma may benefit from a more aggressive treatment approach despite the overall poor prognosis.
